# Influence of Perceptual Discriminability and Spatial Distance on Holistic Processing of Faces

**DOI:** 10.3389/fpsyg.2021.669206

**Published:** 2021-09-08

**Authors:** Chao-Chih Wang, Gary C.-W. Shyi, Peter Kuan-Hao Cheng

**Affiliations:** ^1^School of Education Science, Huizhou University, Huizhou, China; ^2^Department of Psychology, National Chung Cheng University, Chiayi, Taiwan; ^3^Research Center for Education and Mind Sciences, National Tsing Hua University, Hsinchu, Taiwan; ^4^Center for Research in Cognitive Sciences, National Chung Cheng University, Chiayi, Taiwan

**Keywords:** face recognition, perceptual discriminability, holistic processing, composite face task, spatial perception

## Abstract

**Background:** Holistic processing is defined as the perceptual integration of facial features, and plays an important role in face recognition. While researchers recognize the crucial role played by holistic processing in face perception, a complete delineation of the underlying mechanisms is impending. Very few studies have examined the effects of perceptual discrimination and spatial perception on holistic processing. Hence, the present study aimed to examine the influence of perceptual discrimination and spatial perception on face recognition.

**Methods:** We conducted two experiments by manipulating the perceptual discriminability of the target (the top-half faces) and non-target face (the bottom-half faces) parts in the composite-face task and examined how perceptual discriminability may affect holistic processing of faces.

**Results:** The results of Experiment 1 illustrated that holistic processing was modulated by the perceptual discriminability of the face. Furthermore, differential patterns of perceptual discriminability with the target and non-target parts suggested that different mechanisms may be responsible for the influence of target and non-target parts on face perception. The results of Experiment 2 illustrated that holistic processing was modulated by spatial distance between two faces, implicating that feature-by-feature strategy might decrease the magnitude of holistic processing.

**Conclusion:** The results of the present study suggest that holistic processing may lead to augmented perception effect exaggerating the differences between the two faces and may also be affected by the feature-by-feature strategy.

## Introduction

Face recognition is the ability to recognize familiar and unfamiliar human faces (Gauthier et al., 2010). In the past, Galton (1879) was possibly the first to suggest that face recognition was attained through the integration of facial features called holistic processing (Young et al., 1987). Succeeding the findings of Galton, there have been a number of studies supporting the notion of holistic processing (Young et al., 1987; Gauthier et al., 2010; McKone, 2010). Furthermore, a number of studies have demonstrated that face processing can be functionally considered as a module, comprising multiple brain areas, particularly the fusiform face area (e.g., Kanwisher et al., 1997) that is located in the lateral portion of the fusiform gyrus. Using the neurophysiological approach, many studies suggested that inferior temporal cortex is related to holistic face processing (Kobatake et al., 1998; Tsao et al., 2006). Additionally, Tsao and Livingstone (2008) suggested that face detection may be processed by a T-shaped configuration including a pair of eyes, the nose, and the mouth. However, despite many previous studies, the exact mechanism underpinning holistic processing remains an unresolved and contentious issue. For example, while judging whether two faces are identical or not, it is unclear how the presence of the non-target part of a face may affect the judgment of the target part.

Young et al. (1987) developed the composite-face task, which measured the behavioral marker for holistic processing in faces. In their study, they created composite faces of celebrities where the top and bottom halves of a face belonged to different celebrities and were joined to form novel faces (Young et al., 1987). The results revealed that the reaction time for the aligned composite face was longer than that for the unaligned non-composite face, where the top and bottom halves of the face were displaced horizontally from each other. The participants named the top-half of the celebrity faces and were affected by the bottom-half in the composite condition, requiring more time than that in the non-composite condition. In other words, face processing involves integration of all features rather than being a piecemeal process and, therefore, the bottom half would affect the judgment of the participants. Following Young et al.'s study, Hole (1994) reported the composite effect using unfamiliar faces. A pair of unfamiliar, non-celebrity faces were presented simultaneously in each trial and participants judged whether or not the top parts of the displayed faces were identical (Young et al., 1987; Hole, 1994). However, it is unclear whether holistic processing would be affected by the feature-by-feature strategy when the stimuli are presented simultaneously (Hole, 1994; Richler et al., 2009; Wang, 2019). After many studies using the composite face task, the task has become a popular tool for its robustness in assessing the magnitude of holistic processing (Gauthier and Tarr, 1997; de Heering et al., 2008; Gauthier et al., 2010; Rossion, 2013; Chua et al., 2015).

**Figure 1:**
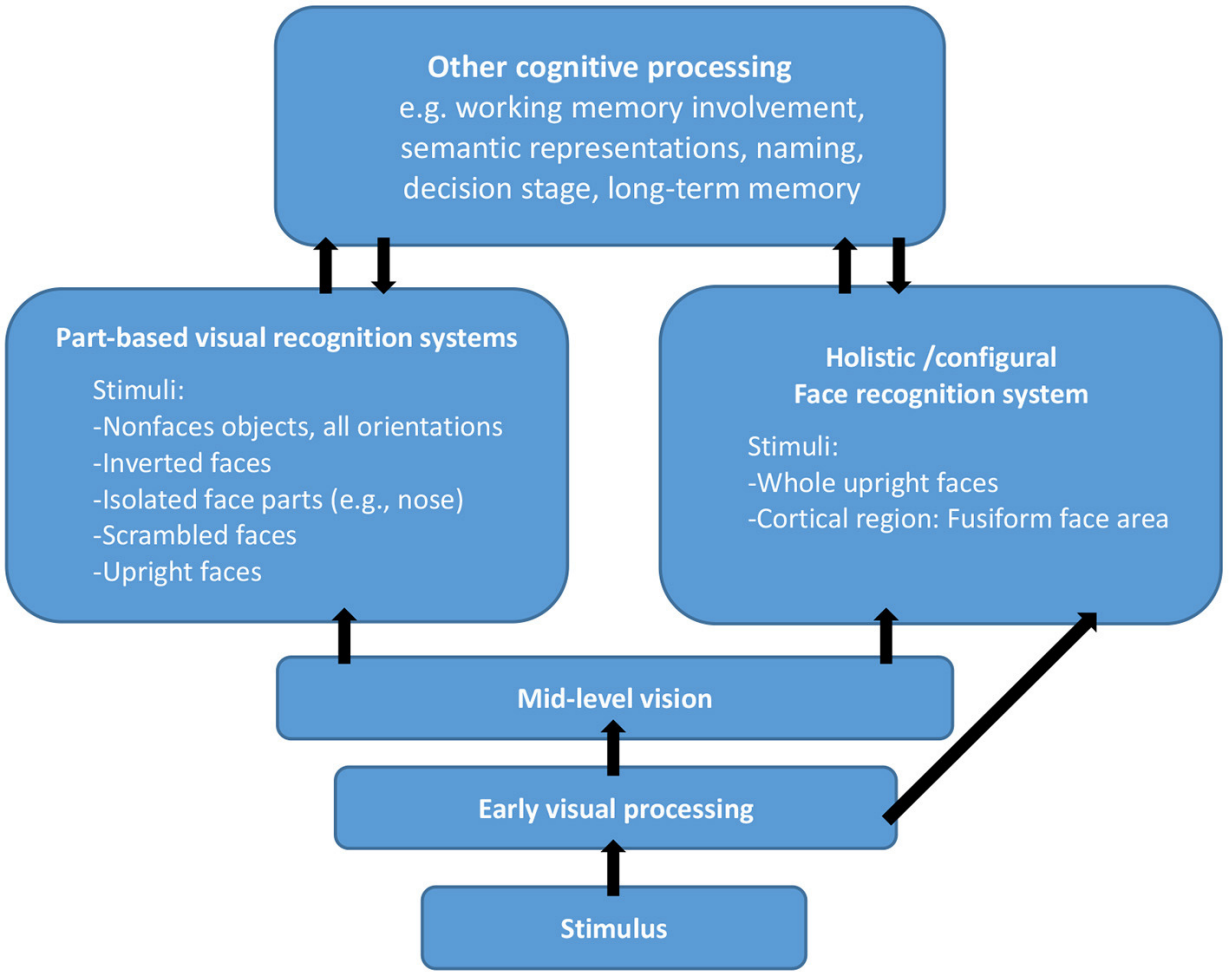
A possible architecture for holistic/configural processing (re-drawn from McKone, 2010).

To elucidate the processing of face recognition, Maurer and colleagues distinguished three types of configural processing: first-order spatial relations, holistic processing, and second-order spatial relations (Maurer et al., 2002). In first-order relations, a face can be detected based on the common template comprising eyes on the top, nose in the middle, and mouth at the bottom. Any visual stimulus with features conforming to that pattern can be perceived as a face, even when features are made of vegetables, such as those in the Arcimboldo paintings. “Holistic processing” is defined as the integration of features. Lastly, Maurer et al. suggested that all faces share the same first-order relations and distinguishing between them requires noticing subtle variations among features. Therefore, second-order relations involve processing of differences in the relations among facial features, especially their spatial relations. However, the ways to make second-order relations detect spatial relations easily is unclear. Following McKone (2010), it may be possible to explain the distinction between part-based and holistic processing (see Figure 1). According to her model, the first step involves early visual processing (e.g., line, shape, and color) and mid-level vision involves further processing on the outputs of early processing. However, it is undetermined if and how mid-level and early visual processing may affect the holistic/configural face recognition system (McKone, 2010) and the factors that might probably affect the holistic processing and part-based processing (Farah et al., 1998).

According to a few studies (Jiang et al., 2011; Goffaux, 2012; Rossion, 2013), holistic processing is affected by the shape of a face. For example, local features were manipulated to attain different discriminability that affected holistic processing (Goffaux, 2012), indicating that holistic processing may be affected by local discriminability. Furthermore, the perception of local discriminability may occur prior to holistic processing. Goffaux and Rossion (2006) used spatial frequency as an independent variable and found that holistic processing relied on low spatial frequency. The local features and spatial frequency may be attributable to the low-level visual processing (see Figure 1), but there have been a few studies that investigated the factors that determine holistic processing. In Goffaux's study (Goffaux, 2012), the similarity of the target face parts were manipulated but failed to manipulate the dissimilarities in the non-target face parts to examine its possible effect on the magnitude of holistic processing. It is important to understand how the target and non-target face parts may interact with each other. In other words, understanding the mechanisms of holistic processing may require examining the impact of perceptual discriminability on the target and non-target parts. Although many studies have focused on holistic processing, it is currently unclear as to which relevant factors affect holistic processing at the stage of mid-level vision (Figure 1). Therefore, in Experiment 1 of the present study, we manipulated the perceptual discriminability of the target and non-target face parts in the composite-face task and examined how perceptual discriminability may affect holistic processing of faces. High and low perceptual discriminabilities were operationally defined by the similarity in rating face parts by the participants.

## General Method

**Figure 2:**
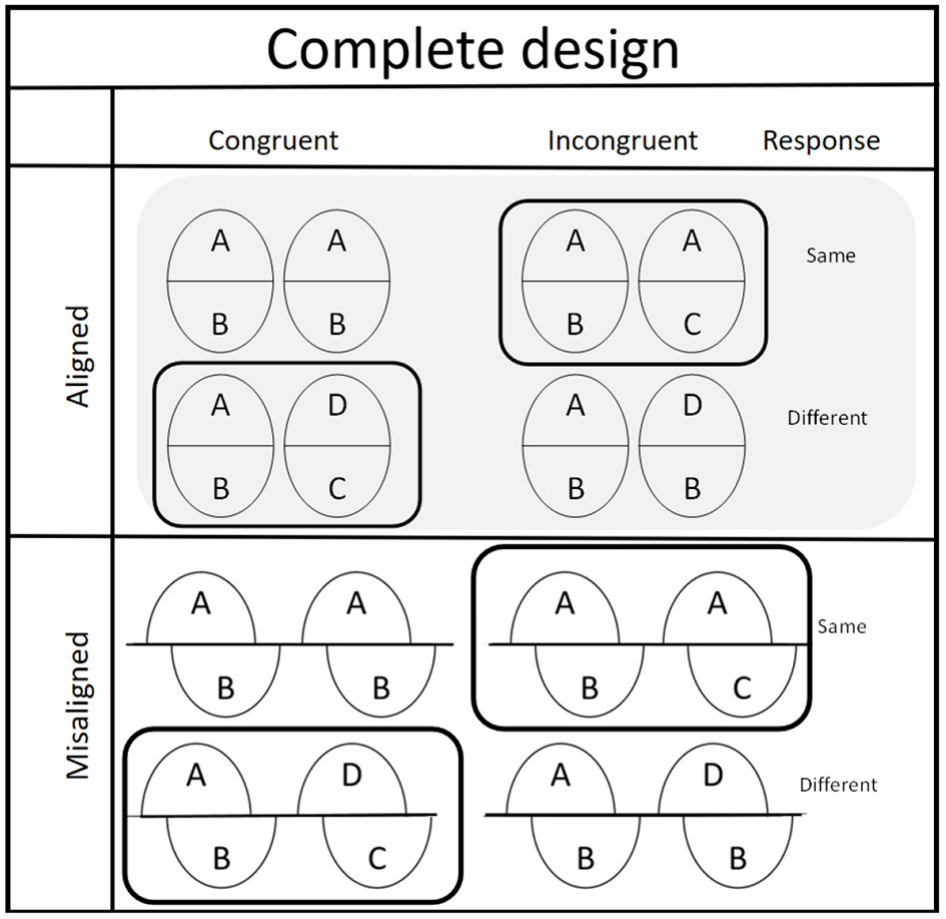
The figure depicting the complete design of composite-face task was redrawn from Gauthier and Bukach (2007).

Gauthier and Bukach (2007) argued that the partial design of composite-face task suffered from only collecting the results of the congruent trials while ignoring those of the incongruent trials. For the incongruent trials, the results may be facilitated with differential magnitude of face perceptual discriminability. Therefore, the present study adopted the complete design of the composite face task (see Figure 2).

When the responses of the target part are congruent with those of the non-target part, these trials belong to the congruent trials. In contrast, in trials where responses from target and non-target parts are incongruent, those trials are as the incongruent trials. The performance in the complete design is sensitivity [*d*' = *Z*(hit) - *Z*(false alarm)] based on the theory of signal detectability (Green and Swets, 1966).

However, the composite effect, entailing an interaction between alignment and congruency, may be obtained using non-face stimuli (e.g., line pattern or line-drawn patterns), suggesting that the effect may include both top–down and bottom–up routes depending on object-based information (Zhao et al., 2016). Therefore, it is better to use the inverted faces as the control group. According to Wang (2019), congruency inversion effect, the dependent measure of holistic processing, was defined as the performance difference of congruency effect, in terms of mean difference scores, collapsed across orientation and congruency. In addition, we also computed outcome performance involving holistic processing and part-based processing, which was defined, respectively, in terms of perceptual discriminability sensitivities in the target and non-target conditions.

## Experiment 1

### Methods

#### Participants

A total of 29 participants (10 men and 19 women) from Kaohsiung Medical University in Kaohsiung, Taiwan, participated in the composite-face task. None of them had been exposed to the composite-face task over the past 3 months (to avoid any lingering learning and/or practice effect). All participants had normal or corrected-to-normal vision, and each received a monetary payment of NT$ 60 (about US$ 2). Each participant spent ~25 min to complete the experiment. All participants were recruited with the approval of the Human Research Ethics Committee of National Chung Cheng University (No. CCUREC104082101). All participants provided informed consent prior to the experiment proper.

#### Stimuli

We generated face stimuli with different perceptual discriminabilities in their relevant (target) and irrelevant (non-target) parts. We created three-dimensional (3-D) pictures of 16 target faces (eight men and eight women) based on images of the Taiwanese face database created by Shyi et al. (2013) using *FaceGen 3.1* (Singular Inversions, Canada). Furthermore, for each target face, we used *FaceGen* to generate 16 new faces with arbitrarily chosen face similarity (40% change). The new faces were generated from multivariate normal distributions for each gender, taken from *FaceGen*'s dataset of 273 high-resolution 3-D scans. We recruited a second group of 20 participants (10 men and 10 women) from the National Chung Cheng University of Chiayi County, Taiwan, and asked them to rate the magnitude of similarity in the face stimuli. Based on the rating results, we selected two sets of stimuli as targets (relevant parts). The first set of stimuli comprised four pairs of two top-half male and female faces with a mean rated similarity of 69.1% as the stimuli of low perceptual discriminability condition. The second set comprised of the same number of pairs of two top-half male and female faces with a mean similarity score of 30.1% as the stimuli of high perceptual discriminability condition. We also chose another two sets of stimuli as irrelevant parts, with similar mean rated similarity of 68.6% as the stimuli of low perceptual discriminability condition and 29.3% as the stimuli of high perceptual discriminability condition.

**Figure 3:**
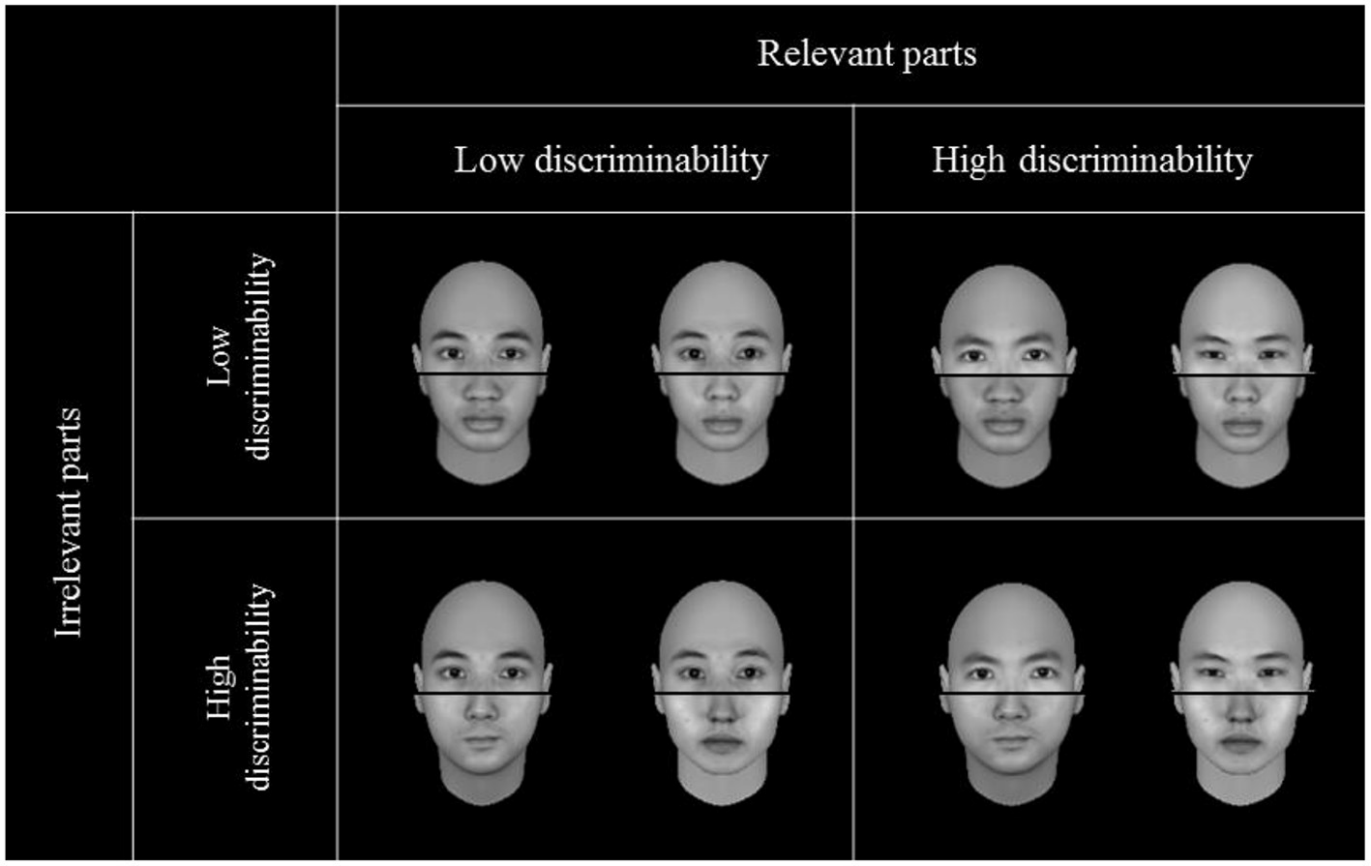
The stimuli include high and low perceptual discriminabilities for relevant and irrelevant parts used in both experiments. Note: the relevant part (or target) always refers to the top half and irrelevant part refers to the bottom half. All the relevant halves and irrelevant halves within a pair were not exactly identical, but the relevant halves on the top two panels were the same as those on the bottom two panels.

In the upright condition, the top half was always considered the relevant (target) part, while the bottom half was always considered the irrelevant (non-target) part. In the inverted condition, the relevant part was the bottom half, and the irrelevant part was the top half (see Figure 3). For both upright and inverted conditions, the relevant parts are those parts with eyes.

When shown on the display screen, each face had a width of ~3.1 cm and a height of 4.4 cm, extending a visual angle of ~3.9° × 6° at a viewing distance of ~45 cm. The distance between the two faces is ~5° apart. An overextended black line was overlaid horizontally in the mid-section of each face to demarcate the top and bottom halves of the face. The line between the two halves was 8.7 cm in length and 0.18 cm in height, extending a visual angle of ~11° × 0.23°. The black overlaid line did not disrupt the perceptual integrity of the face. Its presence was necessary so that participants could unambiguously tell the top apart from the bottom of a face when they were asked to judge whether the top halves of the two presented faces were identical. All stimuli of the composite faces were rendered into grayscale and presented against a dark background on a 15-inch ASUS monitor. The stimuli presentation and responses recording were controlled by *E-Prime* 2.0 (Psychological Software Tools, United States).

#### Design

We adopted the complete composite-face research design and manipulated two factors, perceptual discriminability (high vs. low) and part (relevant vs. irrelevant), as within-subject factors. In the upright condition, the relevant part always referred to the top half of the face, and the irrelevant part referred to the bottom half. In contrast, in the inverted condition, the relevant part always referred to the bottom half of the face, and the irrelevant part referred to the top half.

#### Procedure

In each trial, a “+” sign serving as the fixation point was first shown at the center of the display screen for 500 ms, after which a pair of faces were presented for 2,000 ms, followed by a mask lasting for 200 ms. There were two blocks of trials in Experiment 1: the upright face block and the inverted face block. The order of the two blocks was counterbalanced across participants, where half of the participants saw upright faces in the first block and then saw inverted faces in the second block, and *vice versa*. The spatial distance between the pair of faces was ~5°. The participants were asked to judge whether the relevant (top) halves of the pair of faces were identical, while ignoring the irrelevant (bottom) halves. Each participant completed a total of 256 trials, representing a combination of orientation and perceptual discriminability of relevant and irrelevant parts.

### Results

The mean accuracies for judging the relevant parts (top halves) of face pairs in each condition, in terms of hit rate, correction rejection (CR), and *d*' are listed in Table 1. The mean congruency inversion effects (i.e., the difference in congruency effect between the upright and inverted conditions) as a function of part relevance (relevant vs. irrelevant) and perceptual discriminability (high vs. low) with 2,000 ms of exposure duration are listed in Table 2.

**Table 1:** Descriptive statistics for each condition in Experiment 1 (*N* = 29).

**Perceptual discriminability**			**Upright**			**Inverted**		
**Relevant**	**Irrelevant**	**Congruency**	**HR**	**CR**	***d'***	**HR**	**CR**	***d'***
Low	Low	Congruent	0.96 (0.10)	0.59 (0.21)	2.29 (0.74)	0.91 (0.13)	0.50 (0.28)	1.68 (0.96)
		Incongruent	0.87 (0.15)	0.55 (0.26)	1.59 (1.29)	0.94 (0.09)	0.51 (0.30)	1.91 (1.15)
	High	Congruent	0.91 (0.12)	0.78 (0.19)	2.68 (1.15)	0.92 (0.09)	0.57 (0.22)	1.86 (0.79)
		Incongruent	0.64 (0.23)	0.52 (0.23)	0.59 (0.80)	0.89 (0.15)	0.48 (0.23)	1.50 (0.87)
High	Low	Congruent	0.91 (0.13)	0.98 (0.50)	3.80 (0.88)	0.92 (0.09)	0.95 (0.11)	3.60 (0.94)
		Incongruent	0.86 (0.18)	0.98 (0.40)	3.55 (1.01)	0.92 (0.12)	0.93 (0.10)	3.59 (1.17)
	High	Congruent	0.89 (0.15)	1.00 (0.02)	3.86 (0.89)	0.93 (0.09)	0.93 (0.09)	3.54 (1.07)
		Incongruent	0.67 (0.22)	0.98 (0.04)	2.79 (0.97)	0.92 (0.11)	0.94 (0.10)	3.54 (0.91)

**Table 2:** The mean congruency inversion effect (d') for each condition in Experiment 1 (*N* = 29) (standard deviations are shown in parentheses).

**Perceptual discriminability**		**Orientation**		**Congruency inversion effect**
**R**	**IR**	**Upright**	**Inverted**	
Low	Low	0.7	−0.23	0.93
		(1.22)	(1.2)	(1.48)
	High	2.09	0.36	1.74
		(1.47)	(1.17)	(1.75)
High	Low	0.25	0.01	0.24
		(1.02)	(1.02)	(1.51)
	High	1.07	0.00	1.07
		(1.01)	(1.15)	(1.56)

*R, relevant (top half); IR, irrelevant (bottom half)*.

#### Holistic Processing

The mean congruency inversion effects were submitted to a 2 (relevant-part discriminability: high vs. low) × 2 (irrelevant-part discriminability: high vs. low) two-way repeated-measures ANOVA. The main effect of the relevant-part discriminability was significant, *F*_(1,28)_ = 4.57, *MSE* = 13.34, *p* < 0.05, $\eta_{\text{p}}^{2}$ = 0.14, indicating that when the relevant parts had low perceptual discriminability, the magnitudes of holistic processing revealed by the congruency inversion effect (*M* = 1.33) was greater than that when they were highly discriminable (*M* = 0.65). Likewise, the main effect of the irrelevant part discriminability was also significant, *F*_(1,28)_ = 7.64, *MSE* = 19.38, *p* < 0.05, $\eta_{\text{p}}^{2}$ = 0.214, indicating that when the irrelevant parts had low discriminability, the magnitude of holistic processing (*M* = 0.58) was lower than that when they were highly discriminable (*M* = 1.40). The two-way interaction between relevant and irrelevant part discriminability, however, was not significant, *F* < 1.

**Figure 4:**
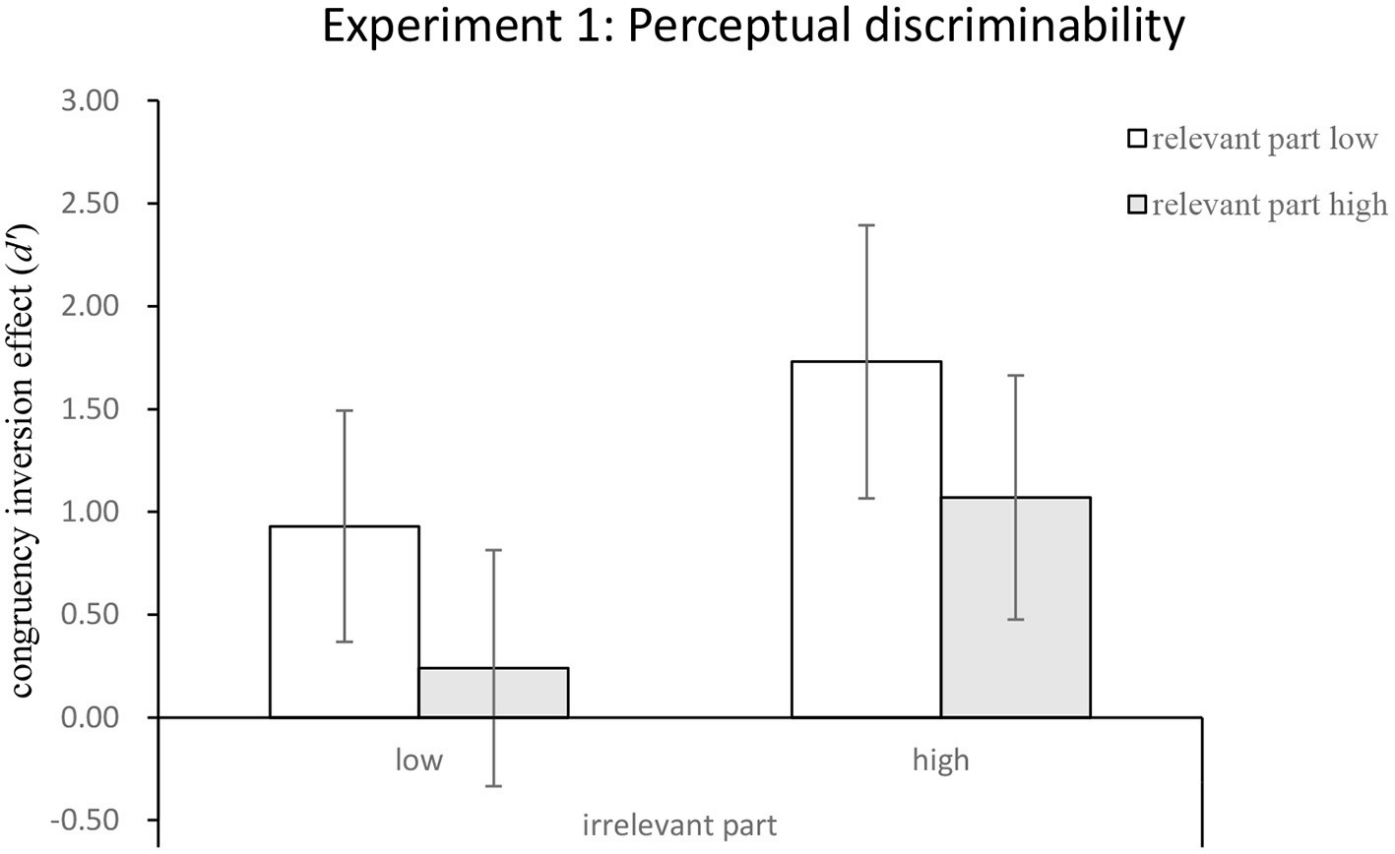
The mean congruency inversion effect for holistic processing as a function of perceptual discriminability of the relevant and irrelevant parts; the error bars indicate a 95% confidence interval, calculated within participants.

We performed one-sample *t*-test against the null effect to examine whether holistic processing would be modulated by perceptual discriminability when the exposure duration was 2,000 ms. We used the Holm–Bonferroni method to control the familywise error rate (α_*B*_ = 0.013, α_*B*_ = 0.017, α_*B*_ = 0.025, and α_*B*_ = 0.05 for the four conditions, respectively). As illustrated in Figure 4, holistic processing was observed when the relevant parts were of low discriminability to each other, regardless of whether or not the irrelevant parts were highly discriminable [low perceptual discriminability for the irrelevant parts: *t*_(28)_ = 3.38, *p* < 0.01 < α_*B*_ = 0.025, *M* = 0.93, 95% CI = (0.37, 1.49); high perceptual discriminability for the irrelevant parts: *t*_(28)_ = 5.36, *p* < 0.001 < α_*B*_ = 0.013, *M* = 1.74, 95% CI = (1.07, 2.40)]. However, holistic processing was observed when the irrelevant parts were highly discriminable under high perceptual discriminability for the relevant parts, *t*_(28)_ = 3.69, *p* < 0.01 < α_*B*_ = 0.017, *M* = 1.07, 95% CI = (0.47, 1.66), but not when irrelevant parts were less discriminable, *t* < 1.

#### Sensitivities of the Irrelevant and Relevant Parts

**Figure 5:**
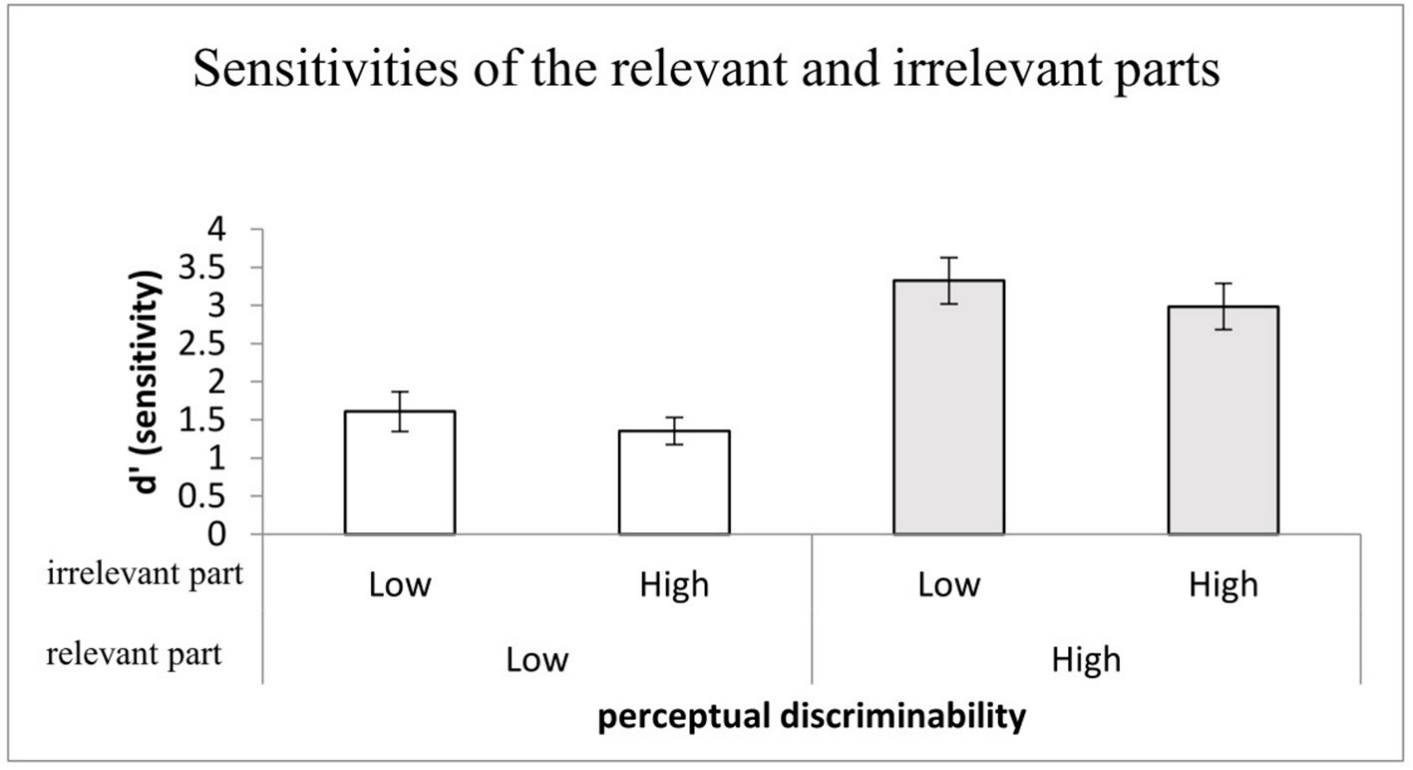
The mean *d'* for perceptual discriminability as a function of the relevant (target) part and irrelevant (non-target) part discriminability; error bars indicate 95% confidence interval calculated within participants.

The identical 2 (the relevant part discriminability: high vs. low) × 2 (the irrelevant part discriminability: high vs. low) repeated-measures ANOVA was conducted for the outcome performance, which was defined as the mean sensitivities collapsed across orientation and congruency (see Figure 5). The main effect of the relevant part discriminability was significant, *F*_(1,28)_ = 160.36, *MSE* = 81.22, *p* < 0.001, $\eta_{\text{p}}^{2}$ = 0.851, reflecting that the mean performance in the high discriminability condition (*M* = 3.15) was better than that in the low discriminability condition (*M* = 1.48). The main effect of the irrelevant part discriminability also was significant, *F*_(1,28)_ = 11.19, *MSE* = 2.58, *p* < 0.01, η_*p*_^2^ = 0.286. Consistent with the relevant target part, the mean performance of the irrelevant part in the high discriminability condition (*M* = 2.47) was better than that in the low discriminability condition (*M* = 2.17). No interaction between perceptual discriminability of the relevant and irrelevant parts was found, *F* < 1. Moreover, in terms of planned comparisons, one-sample *t*-tests showed that the mean performances of the four conditions were all above the chance level, *t*s > 12.6, *p*s < 0.001 < α_*B*_ = 0.013 [relevant vs. irrelevant parts of low perceptual discriminability, *t*_(28)_ = 12.6, *p* < 0.001, *M* = 1.61, 95% CI = (1.35, 1.87); target for low perceptual discriminability vs. the irrelevant part for high perceptual discriminability, *t*_(28)_ = 15.56, *p* < 0.001, *M* = 1.35, 95% CI = (1.18, 1.52); target for high perceptual discriminability with the irrelevant part for low perceptual discriminability *t*_(28)_ = 22.50, *p* < 0.001, *M* = 3.33, 95% CI = (3.02, 3.63); target and the irrelevant part for high perceptual discriminability, *t*_(28)_ = 20.15, *p* < 0.001, *M* = 2.98, 95% CI = (2.68, 3.29)].

### Discussion

The results of Experiment 1 suggest that perceptual discriminability affects holistic processing of human faces, such that the discriminability of relevant and irrelevant parts can make independent contributions to the overall distinctiveness of a face. Interestingly, holistic processing was not evident when the relevant parts were highly discriminable and the irrelevant parts were less discriminable. According to the patterns of the results (see Figure 4), the perceptual discriminability of the relevant and irrelevant parts of a face appeared to provide the opposite effects modulating holistic processing. When the relevant parts were more distinctive owing to their high discriminability, the holistic effect became weaker. In contrast, when the irrelevant parts were under the same distinctive discriminability level, the holistic effect became stronger. Therefore, the present study seems to perceive distinctive faces relying on part-based face recognition systems faster than holistic processing. Furthermore, it implied that feature processing takes less time to judge, compared with holistic processing, consistent with Meinhardt et al. (2014).

However, the relevant parts revealed a stronger effect on holistic processing when they were less discriminable, suggesting that integrating the irrelevant parts through holistic processing may help elevate the distinctiveness of the two different faces. In contrast, when the relevant parts were highly discriminable, less holistic processing was required to distinguish the faces.

In addition to perceptual discriminability, space perception may also affect holistic processing. When participants adopt the feature-by-feature strategy, it is not clear whether the performances will be consistent with the pattern of results from Experiment 1. Although several studies have adopted the composite face presented simultaneously, it has not been investigated whether and how spatial distance between the targets of faces affects holistic processing (Hole, 1994; Wang, 2019). However, some studies have reported inconsistent results because the participants adopted the feature-by-feature strategy (Richler et al., 2009; Wang, 2019). To better understand the role of stimuli exposure differences, we conducted Experiment 2. In other words, to avoid feature-by-feature strategy, spatial distance and exposure duration were manipulated.

## Experiment 2

In Experiment 2, we examined how spatial distance may affect holistic processing. In Experiment 1, the composite faces were presented simultaneously, and it is unclear whether the performances will be the same or different when feature-by-feature strategy is effectively avoided (Hole, 1994; Richler et al., 2009; Wang, 2019). At present, there are only a few studies to examine this issue. To minimize the possibility of feature-by-feature strategy, the exposure time was reduced to 500 ms and spatial distance between the two faces presented was increased (Wang, 2019).

### Methods

#### Participants

A total of 23 participants (6 men and 17 women) from the Southern Taiwan University of Science and Technology participated and none of them had experienced the composite task over the past 3 months. All participants had normal or corrected-to-normal vision, and each received a monetary payment of NT$120 (about US$ 4). Each participant spent ~55 min completing the experiment. As per the procedure of Experiment 1, all participants were recruited with the approval of the Research Ethical Committee of National Chung Cheng University (No. CCUREC104082101). Informed consent was obtained before the experiment.

#### Stimuli

The stimuli used in Experiment 2 were identical to those used and created in Experiment 1 through face photograph ratings rendered by a second subset of participants.

#### Design and Procedure

**Figure 6:**
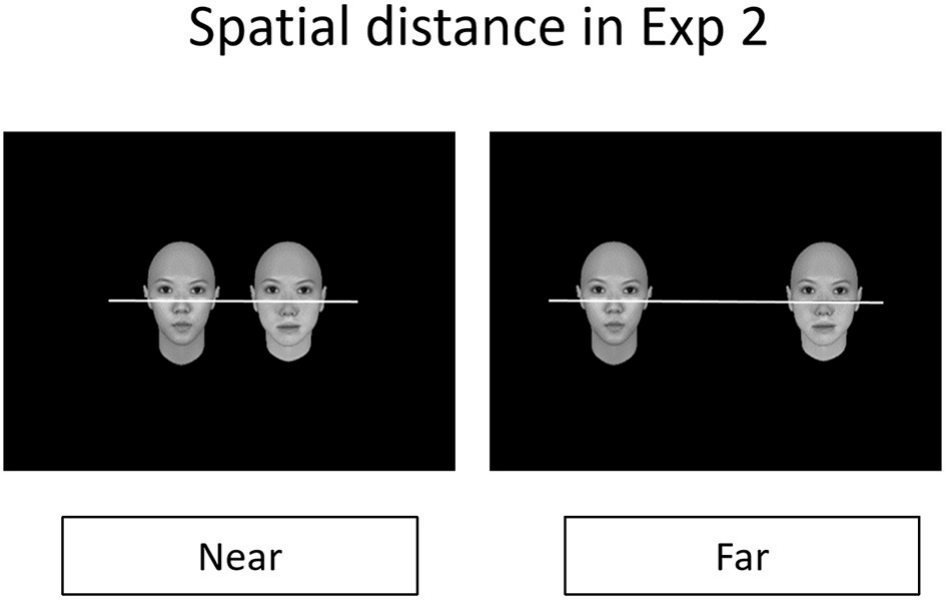
The figure illustrates an example for the display of the stimuli in Experiment 2, where we manipulated the spatial distance in terms of the visual arc between the pair of faces, ~5° for the *near* condition and ~10° for the *far* condition.

We manipulated the perceptual discriminability of both relevant and irrelevant parts as two within-subject factors and manipulated spatial distance between these two faces shown simultaneously (near vs. far) as a within-subject factor (see Figure 6). We used the same dependent measure used in Experiment 1.

In each trial, a “+” sign serving as the fixation point was presented at the center of a display screen for 500 ms, after which a pair of face stimuli were shown for 500 ms, followed by a mask lasting for 200 ms. As in Experiment 1, half of the participants saw upright faces first and then saw inverted faces, and the order was reversed for the other half. The spatial distance in terms of the visual arc between the pair of faces was ~5° for the *near* condition and ~10° for the *far* condition. The two independent variables of orientation (upright vs. inverted) and spacing (near vs. far) were counterbalanced. Half of the participants participated in the near condition first, which included upright and inverted face levels, and then in the far condition that included upright and inverted face levels. The order of participation was reversed for the other half of the participants. The upright and inverted conditions were randomized for the near and far conditions. Each participant completed 512 trials in the second part.

### Results

The mean accuracy of correct identification of the top (relevant) halves in each of the manipulated conditions is listed in Table 3. We calculated the mean congruency inversion effects in each condition in the same way as in Experiment 1, and the results are listed in Table 4.

**Table 3:** Mean hit rate (HR) and correct rejection rate (CR) as a function of the conditions manipulated in Experiment 2 (*N* = 23) (standard deviations are shown in parentheses).

	**Per. discriminability**			**Upright**			**Inverted**		
	**Relevant**	**Irrelevant**	**Congruent**	**HR**	**CR**	***d'***	**HR**	**CR**	***d'***
Near	Low	Low	Congruent	0.85 (0.19)	0.45 (0.23)	1.05 (0.77)	0.86 (0.17)	0.42 (0.26)	0.93 (0.86)
			Incongruent	0.82 (0.21)	0.42 (0.27)	0.85 (0.88)	0.82 (0.22)	0.44 (0.27)	0.91 (0.84)
		High	Congruent	0.86 (0.14)	0.51 (0.30)	1.23 (0.92)	0.85 (0.18)	0.46 (0.26)	1.04 (0.7)
			Incongruent	0.69 (0.28)	0.41 (0.26)	0.33 (0.8)	0.82 (0.22)	0.44 (0.30)	0.94 (0.62)
	High	Low	Congruent	0.90 (0.17)	0.89 (0.10)	2.71 (0.7)	0.88 (0.19)	0.84 (0.16)	2.42 (0.96)
			Incongruent	0.81 (0.24)	0.90 (0.17)	2.47 (1.09)	0.81 (0.24)	0.83 (0.14)	2.18 (0.91)
		High	Congruent	0.86 (0.18)	0.95 (0.09)	2.86 (0.82)	0.82 (0.23)	0.85 (0.14)	2.3 (0.91)
			Incongruent	0.67 (0.27)	0.91 (0.16)	2.02 (1.04)	0.85 (0.22)	0.82 (0.18)	2.32 (1.00)
Far	Low	Low	Congruent	0.88 (0.18)	0.48 (0.32)	1.3 (1.16)	0.81 (0.21)	0.46 (0.33)	0.92 (0.91)
			Incongruent	0.81 (0.23)	0.45 (0.30)	0.89 (1.09)	0.81 (0.18)	0.45 (0.30)	0.94 (0.78)
		High	Congruent	0.85 (0.20)	0.63 (0.27)	1.64 (0.95)	0.81 (0.19)	0.37 (0.24)	0.66 (0.58)
			Incongruent	0.62 (0.28)	0.40 (0.25)	0.11 (0.9)	0.83 (0.22)	0.40 (0.31)	0.81 (0.89)
	High	Low	Congruent	0.88 (0.21)	0.91 (0.15)	2.8 (1.04)	0.84 (0.19)	0.84 (0.17)	2.34 (0.7)
			Incongruent	0.79 (0.25)	0.93 (0.12)	2.54 (1.02)	0.85 (0.21)	0.85 (0.15)	2.4 (0.85)
		High	Congruent	0.90 (0.16)	0.90 (0.15)	2.81 (0.82)	0.82 (0.17)	0.83 (0.14)	2.15 (0.71)
			Incongruent	0.61 (0.27)	0.92 (0.13)	1.79 (0.86)	0.79 (0.15)	0.87 (0.16)	2.18 (0.77)

**Table 4:** The mean congruency inversion effect (d') for each condition in Experiment 2 (*N* = 23) (standard deviations are shown in parentheses).

**Spatial Distance**	**Perceptual discriminability**		**Orientation**		**Congruency inversion effect**
	**R**	**IR**	**Upright**	**Inverted**	
Near	Low	Low	0.2	0.06	0.13
			(1.06)	(1.11)	(1.73)
		High	0.9	0.13	0.77
			(1.3)	(1.11)	(1.38)
	High	Low	0.24	0.35	−0.11
			(0.97)	(1.00)	(1.4)
		High	0.83	−0.03	0.86
			(0.97)	(0.93)	(1.29)
Far	Low	Low	0.4	−0.02	0.43
			(1.23)	(0.97)	(1.37)
		High	1.52	−0.15	1.67
			(1.06)	(0.88)	(1.4)
	High	Low	0.26	−0.06	0.32
			(0.78)	(0.90)	(1.09)
		High	1.02	−0.03	1.05
			(0.85)	(0.90)	(1.34)

**Figure 7:**
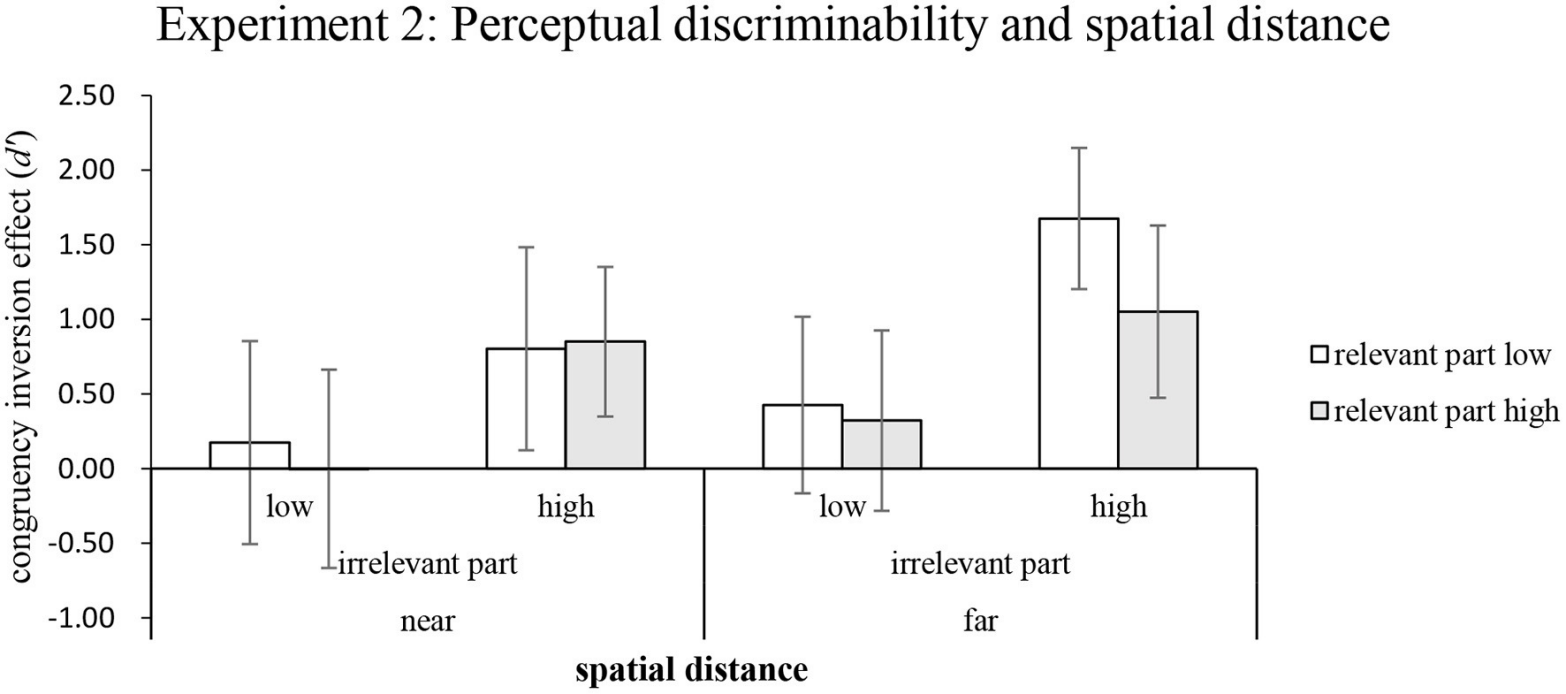
The mean of the congruency inversion effect (*d*') as a function of spatial distance and perceptual discriminability of the relevant and irrelevant parts in Experiment 2. The first two rows of the legend denote the perceptual discriminability of irrelevant parts (high vs. low), and the third row denotes spatial distance between the faces (near vs. far). Error bars indicate 95% confidence interval, calculated within participants.

The mean differences in congruency inversion effects between the upright and inverted conditions were submitted to a three-way repeated-measures ANOVA of 2 (distance: near and far) × 2 (discriminability of relevant parts: high vs. low) × 2 (discriminability of irrelevant parts: high vs. low). The results showed that the main effect of distance was significant, *F*_(1,22)_ = 5.28, *MSE* = 7.81, *p* < 0.05, η_*p*_^2^ = 0.194. As shown in Figure 7, the magnitude of holistic processing was larger when the distance between the relevant pair was *far* (*M* = 0.87) than when it was *near* (*M* = 0.46), which indicates that holistic processing was more likely when pairs of faces were further apart from each other. The main effect of the irrelevant part discriminability was also significant, *F*_(1,22)_ = 14.68, *MSE* = 34.32, *p* < 0.01, η_*p*_^2^ = 0.366, indicating that when the irrelevant parts were highly discriminable, the magnitude of holistic processing (*M* = 1.09) was greater than when the irrelevant parts were of low perceptual discriminability (*M* = 0.23). These findings replicated those of Experiment 1. Finally, rather surprisingly, the main effect of relevant-part discriminability was not significant, *F*_(1,22)_ = 1.36, *MSE* = 2.1, *p* > 0.05, η_*p*_^2^ = 0.058. As shown in Figure 7, the relevant parts yielded approximately the same magnitude of holistic processing, regardless of whether they were highly discriminable from each other (*M*s = 0.56 and 0.76 for high and low discriminabilities, respectively). None of the two-way or three-way interactions were significant, *F*s < 1 or *ps* > 0.05.

One-sample *t*-tests were performed to examine whether holistic processing was modulated by perceptual discriminability and spatial distance. The results showed that holistic processing was not observed when the irrelevant parts had low discriminability, regardless of whether the pair of faces were displayed close to or far away from each other (*t*s < 1 or *p*s > 0.05). In contrast, holistic processing was clearly present when the irrelevant parts were perceptually highly discriminable, regardless of whether the relevant parts were highly discriminable or whether the pair of faces were displayed near or far away from each other: far and relevant parts that were highly discriminable: *t*_(22)_ = 5.75, *p* < 0.001, *M* = 1.67, 95% CI = (1.07, 2.28); far and relevant parts that were less discriminable: *t*_(22)_ = 3.77, *p* = 0.001, *M* = 1.05, 95% CI = (0.47, 1.63); near and relevant parts that were highly discriminable: *t*_(22)_ = 3.52, *p* < 0.01, *M* = 0.85, 95% CI = (0.35, 1.35), and near and relevant parts that were less discriminable: *t*_(22)_ = 2.50, *p* < 0.05, *M* = 0.80, 95% CI = (0.14, 1.47)].

**Figure 8:**
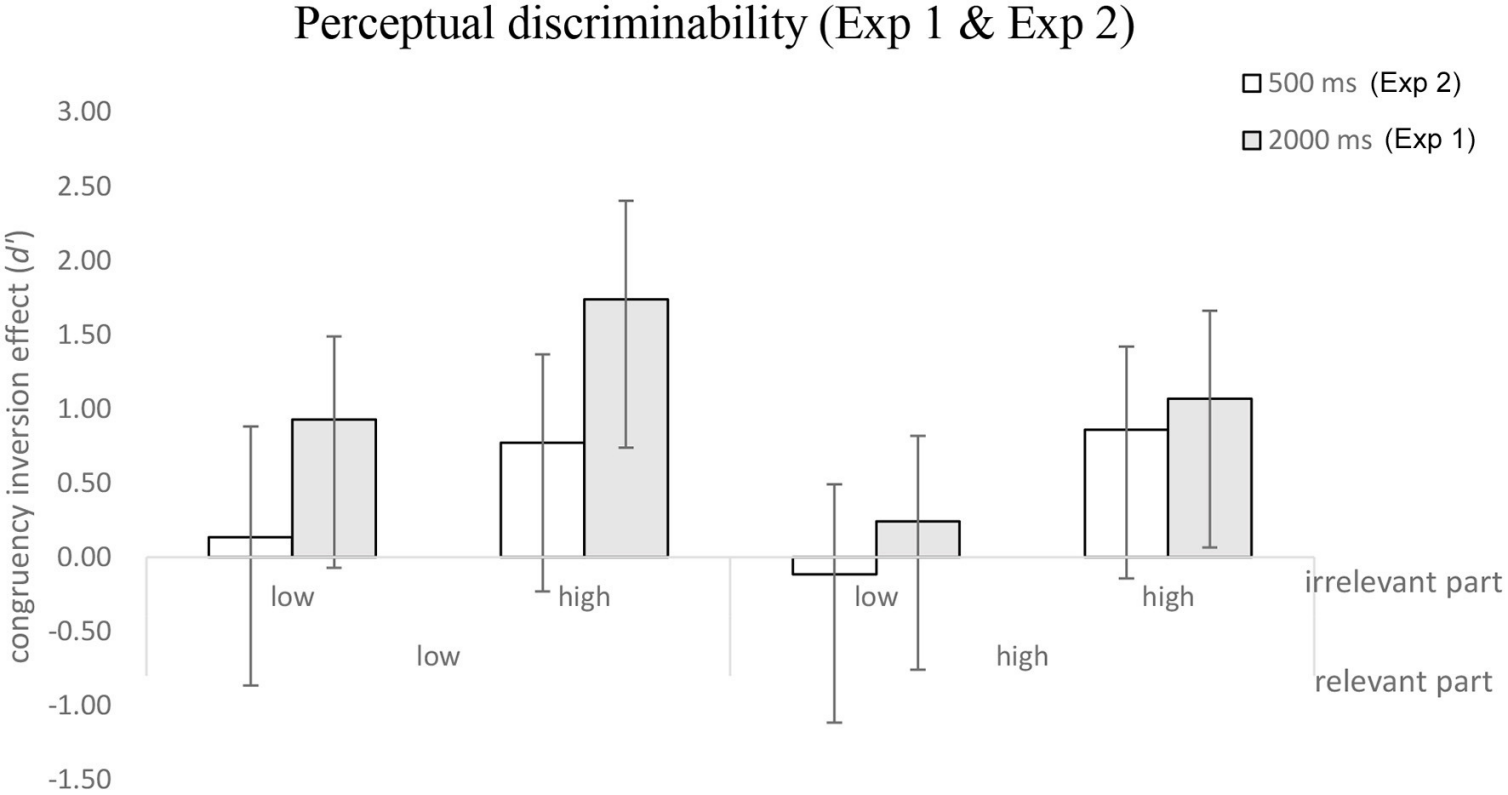
The mean of the congruency inversion effect (*d*') as a function of exposure (Experiments 1 and 2) and perceptual discriminability of the relevant and irrelevant parts. Error bars indicate 95% confidence interval, calculated within participants.

To clarify how stimuli exposure time might have modulated holistic processing, a 2 (the perceptual discriminability of relevant parts: high vs. low) × 2 (perceptual discriminability of irrelevant parts: high vs. low) × 2 (exposure time: 500 ms and 2,000 ms) mixed repeated-measures ANOVA was conducted on the congruency inversion effect. As shown in Figure 8, the results showed that the effect of exposure was significant [*F*_(1,50)_ = 6.3, *MSE* = 17.36, *p* < 0.05, η_*p*_^2^ = 0.112], and that the main effect of the irrelevant part was also significant [*F*_(1,50)_ = 14.19, *MSE* = 33.76, *p* < 0.001, η_*p*_^2^ = 0.221]. There were neither other significant main effects nor significant interactions, *F*s < 1 or *p*s > 0.05.

**Figure 9:**
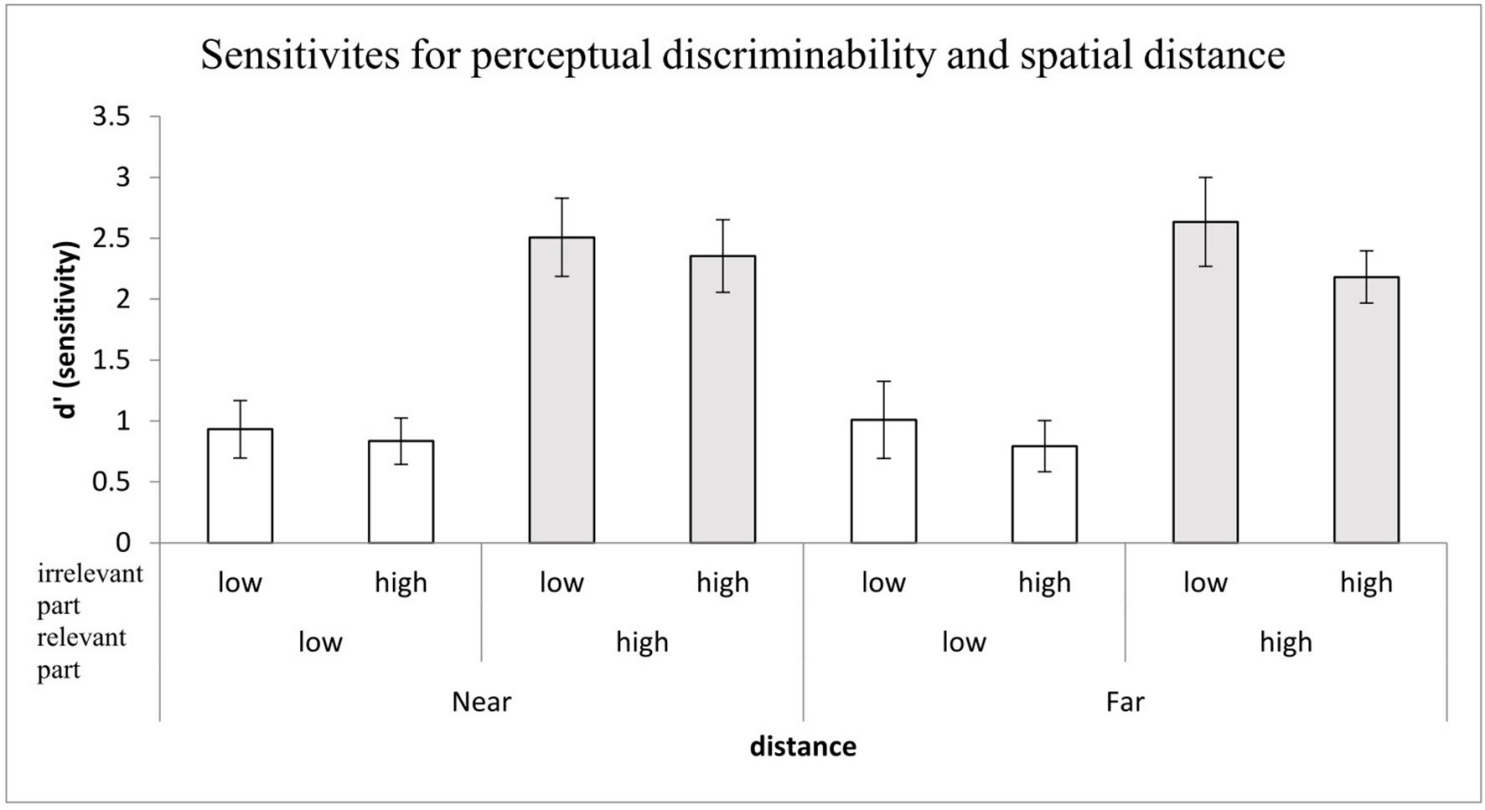
The mean sensitivities collapsed across orientation (*d*') as a function of perceptual discriminability of the relevant and irrelevant parts. Error bars indicate 95% confidence interval, calculated within participants.

As shown in Figure 9, the mean sensitivities (*d*') collapsed across orientation (upright and inverted) and congruency (congruent and incongruent) were submitted to the same 2 × 2 × 2 repeated-measures ANOVA for holistic processing analyses. The main effect of the relevant part discriminability was significant, *F*_(1,22)_ = 172.18, *MSE* = 107.3, *p* < 0.001, η_*p*_^2^ = 0.887, indicating that performances for relevant parts that were highly discriminable (*M* = 2.42) were better than those that were highly similar (i.e., of low discriminability) (*M* = 0.89). Likewise, the main effect of the irrelevant part discriminability was significant, *F*_(1,22)_ = 10.5, *MSE* = 2.42, *p* < 0.01, $\eta_{\text{p}}^{2}$ = 0.323, indicating that performances for irrelevant parts that were highly discriminable (*M* = 1.54) were worse than those that were highly similar (*M* = 1.77). Finally, the main effect of distance between the pair of faces was not significant, *F* < 1; neither were any of the two-way or three-way interactions, *F*s < 2.36 or *p*s > 0.05.

One-sample *t*-tests as planned comparisons were performed to see whether performance in each condition was better than the chance (i.e., *d*' = 0). The results revealed, as shown in Figure 9, that performances in all eight conditions representing the 2 × 2 × 2 combinations of distance, relevant part discriminability, and irrelevant part discriminability were all significantly different from chance, *t*s > 6.59, or *p*s < 0.001 < α_*B*_ = 0.004.

### Discussion

The results of Experiment 2 indicated that the performances of the congruent trials were better than those of the incongruent trials in the upright condition, but not in the inverted condition (see Table 4). These results are consistent with Experiment 1 and many previous studies that have adopted the complete design. Furthermore, the performances in Experiment 2 were better in the high discriminability conditions than those in the low discriminability conditions, which were also consistent with the findings in Experiment 1 (see Table 4). Additionally, the results revealed that spatial distance affects holistic processing and suggest that feature-by-feature strategy reduces holistic processing in the near condition.

The magnitudes of the composite inversion effects in Experiment 2 were smaller than those in Experiment 1 (Figure 7). As a result, we conducted a comparison of the performances at 500 and 2,000 ms between the two experiments through trials in which the faces were presented at an equal distance (5°) in both the experiments (see Figure 8). The results indicated that the magnitudes of holistic processing were evident when the exposure time was reduced, particularly in the high discriminability condition of irrelevant parts. Our findings, which are consistent with a few studies (Richler et al., 2009, 2011, 2012), suggest that the magnitude of holistic processing may decrease or even disappear as the exposure time declines under specific conditions (low perceptual relevant part and low perceptual irrelevant part) (see Figure 8). In other words, it appears to be necessary to have a sufficient exposure time to obtain the composite inversion effect as evidence for holistic processing (Wang, 2019).

In addition, holistic processing is also affected by spatial distance, which may increase when the distance between the targets is in the far condition (see Figure 7). There are two possible explanations for these results. First, we speculate that holistic processing is related to eye movements. Second, the spatial factor may also have affected the performances for inverted faces and resulted in a rather larger composite inversion effect. These two hypotheses need to be further tested.

## Summary and Overall Discussion

In Experiment 1, while perceptual discriminability of both relevant and irrelevant parts affected holistic processing, their congruency inversion effects were in the opposite direction. However, this opposing effect was missing in Experiment 2. When the exposure duration was reduced from 2,000 ms in Experiment 1 to 500 ms in Experiment 2, different patterns of modulation by perceptual discriminability on holistic processing emerged. Specifically, it appears that the irrelevant parts are more dominant in affecting holistic processing in the early stage because the effects of the irrelevant parts were obtained when exposure duration was reduced to 500 ms in Experiment 2.

Perceptual discriminability of the irrelevant parts has a vastly different effect on holistic processing such that when irrelevant parts are highly discriminable, they help distinguish displayed faces by integrating with the relevant parts through holistic processing. Therefore, perceptual discriminability, presumably a part of low-level perceptual processing, affects holistic processing.

The inversion effect of faces is an important phenomenon in understanding the mechanisms of face processing (Yin, 1969). Several studies interpreted the inversion effect because inverted faces are distorted or disrupted as perceiving inverted faces (Bartlett and Searcy, 1993; Maurer et al., 2002). Furthermore, upright faces, as perceived by human beings, are more accurate because we can perceive them in a proper, ecologically representative manner (Maurer et al., 2002). However, our results suggest an opposite alternative where upright faces may be more accurately perceived than inverted faces because holistic processing would exaggerate the differential features in upright faces, rather than perceiving them precisely. It appears that there is a similar expertise effect for word recognition that would exaggerate the differences of similar words (e.g., boy vs. toy) as we become reading experts (Dehaene, 2010). Thus, we found the face composite illusion according to both experiments in the present study. We have a reason to suspect that face composite illusion facilitates face recognition because the mechanisms of face processing may magnify the differences of similar faces (Tanaka et al., 1998). Our findings suggest that the controversies about the expertise hypothesis and the domain-specificity hypothesis must be re-evaluated because many studies did not control the perceptual discriminability of the stimuli (Carey and Diamond, 1994; Gauthier and Tarr, 2002; Robbins and McKone, 2007).

In conclusion, our findings demonstrated that perceptual discriminability and spatial distance affect the holistic processing of faces directly. Furthermore, the present study demonstrated that the relevant and irrelevant parts of faces have opposite effects on holistic processing. This discrepancy implies that there may be different mechanisms for holistic processing based on the relevant and irrelevant parts.

## Data Availability Statement

The original contributions presented in the study are included in the article/supplementary material, further inquiries can be directed to the corresponding author/s.

## Ethics Statement

The studies involving human participants were reviewed and approved by the Research Ethical Committee of National Chung Cheng University (No. CCUREC104082101). The patients/participants provided their written informed consent to participate in this study.

## Author Contributions

C-CW and PC contributed to the design and rationale of the whole experiments. C-CW and GS analyzed the data and drafted the manuscript. GS was involved in data interpretation. All authors were involved in the revision of the manuscript.

## Funding

The study was supported by The Professorial and Doctoral Scientific Research Foundation of Huizhou University, China (19-15802) and Ministry of Science and Technology, Taiwan (MOST 108-2634-F-007-007) and (109-2410-H-194-039-MY2).

## Conflict of Interest

The authors declare that the research was conducted in the absence of any commercial or financial relationships that could be construed as a potential conflict of interest.

## Publisher's Note

All claims expressed in this article are solely those of the authors and do not necessarily represent those of their affiliated organizations, or those of the publisher, the editors and the reviewers. Any product that may be evaluated in this article, or claim that may be made by its manufacturer, is not guaranteed or endorsed by the publisher.
